# Beyond survival: Multisystem long-term outcomes following HSCT in chronic granulomatous disease

**DOI:** 10.70962/jhi.20250076

**Published:** 2026-02-06

**Authors:** Valentina Guarnieri, Jesmeen Maimaris, Sohilla Lotfy, Elizabeth Rivers, Stuart Adams, Kimberly Gilmour, Andrea Meinhardt, Arnold Awuah, Mercedes Selby, Daisy Shillingford, Emma Gravett, Susanne Kricke Orszulik, Waseem Qasim, David Goldblatt, Helen Braggins, Robert Chiesa, Maaike Kusters, Reem Elfeky

**Affiliations:** 1 Infection, Immunity, and Inflammation Department, Great Ormond Street (GOS) Hospital for Children NHS Foundation Trust, University College London GOS Institute of Child Health, and NIHR GOSH Biomedical Research Centre, London, UK; 2 Department of Health Sciences, University of Florence, Florence, Italy; 3 Division of Immunology, Department of Pediatrics, Faculty of Medicine, Cairo University, Cairo, Egypt; 4 Immunology Department, Great Ormond Street Hospital, London, UK; 5 SIHMDS-Haematology, Great Ormond Street Hospital for Children NHS Foundation Trust, London, UK; 6 University College London Great Ormond Street Institute of Child Health, London, UK; 7 Department of Pediatric Hematology, Oncology and Immunodeficiencies, University Children´s Hospital Giessen, Giessen, Germany; 8 Blood and Bone Marrow Transplant Department, Great Ormond Street Hospital, London, UK

## Abstract

Data on late complications after hematopoietic stem cell transplantation (HSCT) in children with chronic granulomatous disease (CGD) are limited. We retrospectively analyzed 42 pediatric CGD patients who survived >2 years post-HSCT (1994–2020) at Great Ormond Street Hospital. X-linked CGD accounted for 69%. Pre-HSCT comorbidities included lung disease (48%), colitis/fistulizing disease (45%), and liver abscesses (19%). Median HSCT age was 6 years. 10-year overall survival was 95.2%, and event-free survival (events: death, second intervention) was 81%. 13 (33%) cases developed autoimmunity (AI); eight had relapsing or late-onset (>2 years) AI. Organ-specific late effects included lung abnormalities (*n* = 7), liver dysfunction (*n* = 7), renal disease (*n* = 3), and recurrent/new-onset colitis (*n* = 3). Endocrine sequelae comprised obesity (*n* = 15), gonadal dysfunction (*n* = 9), and short stature (*n* = 7). Skeletal (*n* = 6), neurological (*n* = 6), psychological (*n* = 19), visual (*n* = 5), and hearing impairments (*n* = 4) were noted. Late malignancy was reported in two cases. HSCT offers excellent long-term survival in CGD, but significant late effects underscore the need for extended multidisciplinary follow-up.

## Teaser

Guarnieri et al. report long-term outcomes after HSCT in children with chronic granulomatous disease. While survival is excellent, a wide range of autoimmune and organ-specific late effects emerge, highlighting the significance of extended multidisciplinary follow-up to optimize patient care.

## Introduction

Chronic granulomatous disease (CGD) is a rare primary immunodeficiency disorder caused by pathogenic variants in one of six subunits of the nicotinamide adenine dinucleotide phosphate (NADPH) oxidase complex. This enzyme is pivotal for phagocytic cells to generate reactive oxygen species, crucial in pathogen elimination, particularly against catalase-positive bacteria, fungi, and mycobacteria (1).

X-linked (XL) mutations in gp91phox account for approximately two-thirds of cases (2, 3), while autosomal recessive (AR) forms result from mutations in p47, p67, p22, and p40 subunits and EROS (4, 5, 6, 7, 8).

In addition to infections, a hallmark of CGD is failure of clearance of dying immune cells with activation of inflammasomes and suppression of autophagy, leading to the development of hyperinflammatory manifestations in the context of infections, including granulomas, which can affect multiple organs and can cause inflammatory bowel–like disease (IBD) (9).

Conservative management of CGD focuses on lifelong antimicrobial prophylaxis, prolonged courses of antimicrobial treatments, and aggressive treatments of inflammatory complications, often requiring immunomodulatory therapy. In many cases, surgical procedures are required to manage infection-related complications (10, 11). Despite advances in conservative management, long-term survival remains compromised, with ∼50–55% survival by the fourth decade of life and disease-associated complications affecting organ function, growth, and negatively impacting quality of life in survivors (12, 13).

Allogeneic hematopoietic stem cell transplantation (HSCT) offers a curative option for CGD, addressing both infectious and inflammatory manifestations (14, 15, 16). A landmark multicenter study by Chiesa et al. (17) demonstrated excellent survival rates, particularly in pediatric patients receiving well-matched donor grafts. These findings support early HSCT when suitable donors are available.

As more CGD patients survive into adulthood, long-term follow-up of HSCT late effects and outcomes have become increasingly relevant. However, comprehensive long-term outcome data in pediatric CGD patients after HSCT remain limited.

Great Ormond Street Hospital (GOSH) is one of two supra-regional UK centers delivering HSCT for children with inborn errors of immunity (IEIs). A structured long-term follow-up program is in place, allowing for systematic monitoring and data collection over time after HSCT. This study analyzes the long-term outcomes of pediatric CGD patients who underwent HSCT at GOSH between 1994 and 2020.

## Results

### Patient demographics

The study cohort comprised 42 patients (35 males, 83.3%; 7 females, 16.7%) who underwent HSCT for CGD (Tables 1 and 2). The median age at HSCT was 6 years (range 1–15 years). At final follow-up, patients had reached a median age of 16 years (range 6–22 years), with a median posttransplant follow-up duration of 8 years (range 2.4–13 years). Regarding CGD subtypes, XL-CGD (gp91phox deficiency) was predominant, affecting 29 patients (69.0%). The remaining 13 patients presented with AR forms: p47phox deficiency (*n* = 8, 19.0%), p67phox deficiency (*n* = 3, 7.1%), and p22phox deficiency (*n* = 2, 4.8%). A positive family history of CGD was documented in four patients (P26, P32, P38, and P39; 9.5%).

**Table 1.** Patient demographics and transplant characteristics

Patient demographic	No. (%)
Sex	
Male	35 (83.3%)
Female	7 (16.7%)
Median age in years at first HSCT (range)	6 (0.9–15.2 years)
Median age in years at last follow-up (range)	16.8 (6.0–22.0 years)
Median follow-up time post first HSCT in years (range)	8 (2.4–13.8 years)
CGD diagnosis	42
XL-CGD	29 (69.0%)
p47	8 (19.0%)
p67	3 (7.1%)
p22	2 (4.8%)
Number of interventions	45
One HSCT	42 (93.3%)
Post-HSCT DLI	3 (6.6%)
DLI followed by second HSCT	2 (4.4%)
Post-HSCT gene therapy	1 (2.2%)
Donor	45
MUD	23 (51.1%)
MSD	11 (24.4%)
MMUD	10 (22.2%)
MFD	1 (2.2%)
Conditioning intensity	45
Myeloablative (MAC)	11 (24.4%)
—Bu/Cy	6 (13.3%)
—Bu/Flu	3 (6.**7%**)
—Treo/Thio/Flu	2 (4.4%)
Reduced intensity (RIC)	34 (75.6%)
—Flu/Bu	20 (44.4%)
—Flu/Treo	12 (26.**7**%)
—Flu/Melph	1 (2.2%)
—Bu/Cy	1 (2.2%)
Transplant cells	45
Bone marrow	25 (55.5%)
PBSC	18 (40.0%)
Cord	2 (4.4%)
GvHD prophylaxis	45
CSA + MMF	35 (77.8%)
CSA + MTX	6 (13.3%)
CSA + Methylprednisolone	1 (2.2%)
CSA only	2 (4.4%)
None	1 (2.2%)

Patients (*n* = 42) and transplants (*n* = 45). Bu, busulfan; CSA, ciclosporin; Cy, cyclophosphamide; Flu, fludarabine; Melph, melphalan; MFD, matched family donor; MMF, mycophenolate; MTX, methotrexate; PBSC, peripheral blood stem cell; Thio, thiotepa; Treo, treosulfan.

**Table 2.** Patient details, clinical presentation, HSCT details, and complications after HSCT (>2 years)

Pt.	Age at HSCT (years)	Sex	CGD type	Clinical presentation	Donor	Conditioning	aGvHD	cGvHD	Event	Long-term complications (Persistent—P/Transient—T/Intermittent—I)	Long-term complications present at last follow-up/time at last follow-up after HSCT (year)
1	12	M	gp91	− Liver abscess − CGD colitis − Recurrent mouth ulcers − Anal fissures − Balanitis/phimosis	MMUD	Camp Flu Bu	No	No	​	− Mild hypertransaminasemia (T) − Upper limb hypermobility (T) − Recurrent infections (T) − Underweight (P) − Anxiety with PTSD (P) − Depression (P) − OCD tendency (P)	− Underweight − OCD tendency 7.4 years
2	10	M	gp91	− Lung infections (probably fungus) − CGD colitis	MUD	Camp Flu Bu	Grade I gut	No	​	− Overweight (T) − Ophthalmic VZV (T) − Abdominal pain (I) − Dyslipidemia-TG (P) − Chronic neuralgic pain post VZV (P) − Anxiety and panic disorder (P)	− Dyslipidemia-TG − Chronic neuralgic pain after VZV − Anxiety and panic disorder 8 years
3	6	F	p47	− CGD colitis, − Skin granulomas − Lung granulomas, mouth ulcers	MSD	ATG Bu Flu	Grade I gut	No	​	− Abdominal pain (T) − Overweight (T) − Dry skin (T) − Chronic EBV-viremia low levels (P) − Delayed puberty (P) − Anxiety (P)	− Chronic EBV viremia low levels − Delayed puberty − Anxiety 9.7 years
4	5	M	gp91	− Fungal lung infection − Severe LRTI	MUD	Camp Flu Bu	Grade I skin	No	​	− Hypothyroidism (T) − Mild conductive HL (T) − Obesity (P) − Needle phobia (P) − Malocclusion (P)	− Obesity − Needle phobia− Malocclusion 7.7 years
5	10	M	p47	− CGD colitis − Mouth ulcers	MSD	ATG Bu Flu	No	No	​	− Dyslipidemia-TG (P) − Obesity/Overweight (P) − Anxiety-tic disorder (related to ASD pre-BMT) (P)	− Overweight 6.7 years
6	2	M	gp91	− Recurrent LRTI − Recurrent fever − Lymphadenitis	MFD	Camp Flu Bu	Grade I gut and skin	No	​	− Chronic abdominal pain (I) − Mild hypertransaminasemia (P) − Genu valgum (P) − Overweight (P)	− Chronic abdominal pain − Mild hypertransaminasemia − Genu valgum − Overweight 9.8 years
7	3	M	gp91	− Liver abscess − Skin abscess − CGD colitis − Extrinsic allergic alveolitis	MMUD	Camp Flu Bu	Grade II skin	No	​	− Adrenal insufficiency (T) − Premature ventricular tachycardia (T) − Autoimmune hypothyroidism (P) − Overweight (P) − Atopic dermatitis (P)	− Autoimmune hypothyroidism − Overweight − Atopic dermatitis 9.1 years
8	1	M	gp91	− Invasive aspergillosis with lung involvement − BCG-osis	MUD	Camp Flu Treo	Grade II gut	No	​	− Acute viral hepatitis (T) − Dyslipidemia (T) − Tibia fracture (T) − Chronic relapsing AIHA (P) − Residual CT chest changes (P) − Adrenal insufficiency (P) − Short stature (P) − Underweight (P)	− Chronic relapsing AIHA − Residual CT chest changes − Adrenal insufficiency − Short stature − Underweight 9 years
9	4	M	gp91	− Recurrent fevers	MMUD	Camp Flu Bu	Grade I skin	Extensive gut	Died at 8 years secondary to BK nephropathy	− Chronic EBV viremia (T) − Dyslipidemia (T) − Osteopenia (T) − NEC/Intestinal failure (P) − HTN (P) − Hypertransaminasemia (P) − Chronic kidney disease (P) − Obesity/Overweight (P) − Short stature (P) − Frequent skin lesions (P)	− NEC/Intestinal failure − HTN − Hypertransaminasemia − Chronic kidney disease − Overweight − Short stature − Frequent skin lesions 4.7 years
10	11	M	gp91	− Aspergillus LRTI − NTM LRTI − CGD colitis − Perianal fistula	MUD	Camp Bu Cy	No	Limited skin after second HSCT	DLI second HSCT	− Recurrent colitis prior to second HSCT (T)	None 7.6 years
11	5	F	p47	− Fungal CNS infection − Recurrent LRTI with lung nodules − Pericardial effusion	MSD	Bu Flu	No	No	​	− Chronic EBV viremia (T) − Adrenal insufficiency (T) − Anxiety with school refusal (T) − Residual CT chest changes (P) − Hyper-transaminitis (P) − Hypothyroidism (P) − Radio ulnar synostosis (P) − Genu valgum (noted at last visit) − Secondary Amenorrhea-ovarian failure (P) − Focal epilepsy (P) − Cataract (P) − Abnormal teeth structure (P) − Atopic dermatitis (P)	− Residual CT chest changes − Hyper-GGT − Hypothyroidism − Radio ulnar synostosis − Genu valgum − Secondary amenorrhea-ovarian failure − Focal epilepsy − Cataract − Abnormal teeth structure − Atopic dermatitis 13.2 years
12	3	M	p67	− CGD colitis, − Osteomyelitis	MUD	Camp Flu Treo	No	No	DLIsecond HSCT	− GBS-like (T) − Bronchial asthma (P) − Chronic abdominal pain (P) − GH deficiency (P) − Eczema (P) − Alopecia (P)	− Bronchial asthma − Chronic abdominal pain − GH deficiency − Eczema (P) − Alopecia (P) 12.8 years
13	11	M	gp91	− Liver abscess − Retroperitoneal mass	MUD	Camp Flu Treo	No	No	​	− Skin VZV (T)	None 6.5 years
14	9	M	gp91	− CGD colitis − Rectosigmoiditis − Anal fissure − Multiple retinal lesions	MSD	Bu Cy	Grade III skin, liver and gut	No	​	− 2 fractures (T) − HTN (P) − Low bone density (P) − School difficulties (P)	− HTN − Low bone density − School difficulties 9.4 years
15	5	M	gp91	− CGD colitis − Urethral granuloma	MSD	Bu Cy	No	No	​	− Enuresis (T) − Obesity (T) − Testicular disorder (T) − Papillary blepharon-conjunctivitis (T) − Frontal headache (I) − Forgetfulness (P) − White matter changes on CT scan associated with cognitive dysfunction (P)	− Frontal headache − Forgetfulness − White matter changes on CT scan associated with cognitive dysfunction 13.8 years
16	10	M	p22	− CGD colitis − Severe chest infection − Skin granuloma	MUD	Camp Flu Bu	Grade I skin	Gut	​	− Dyslipidemia-TG (T) − Chronic EBV viremia (T) − HSV1 reactivation (T) − New and persistent CT chest changes (P) − School difficulties and bullying (pre- and after HSCT) (P)	− Residual CT chest changes 4.8 years
17	6	M	gp91	− Recurrent lymphadenitis − Recurrent pancreatitis, − Liver abscess − Pneumonia (*Actinomyces*) − Sepsis (*chelonae*) − ITP	MMUD	ATG Bu Flu	Pericardial effusion	No	​	− Hyperglycemia (T) − School difficulties and bullying (T) − Glycosuria (at last visit) − Residual CT chest changes (P) − HTN (P) − Dyslipidemia (P) − Immune complex glomerulonephritis with chronic kidney disease stage III (P) − Cataract (P) − SNHL (P)	− Residual CT chest changes − HTN − Dyslipidemia − Glycosuria − Immune complex glomerulonephritis with chronic kidney disease stage III − Cataract − SNHL 12.2 years
18	15	M	gp91	− CGD colitis, − Eczema − Cerebral aspergillosis	MMUD	Camp Flu Treo	No	No	Deceased following myocardial infarction at 26 years	− Scoliosis (P) − Mild acne and dermatitis (P)	− Scoliosis (P) − Mild acne and dermatitis (P) 10.4 years
19	5	M	gp91	− Aspergillus LRTI − Buttock abscesses − Viral meningitis − CGD colitis − McLeod syndrome	MUD	Camp Flu Bu	Grade I skin	No	​	− Conjunctivitis (T) − Choroidal naevus (at last follow up) − Residual CT chest changes (P) − Overcrowded teeth (P) − Face hyperpigmentation (P)	− Residual CT chest changes − Choroidal naevus − Overcrowded teeth − Face hyperpigmentation 11.3 years
20	9	F	p47	− Skin abscesses − Perianal abscesses − Recurrent URTI and OMA − Asthma	MUD	Camp Flu Bu	Grade II skin	No	​	− Chronic viremia EBV (T) − Chicken pox (T) − Loss of scalp hair (at last visit) − Overweight/Obesity (P) − Ovarian failure (P) − Anxiety and needle phobia (P) − Acne (P)	− Obesity − Ovarian failure − Anxiety and needle phobia − Acne − Loss of scalp hair 10.1 years
21	4	M	gp91	− Recurrent chest infections	MUD	Camp Flu Melph	Grade II skin and gut	Skin and eyes scleroderma after second HSCT	Gene therapy second HSCT	− Polyarthritis (T) − Residual CT chest changes with new scars (P) − SNHL (P)	− Residual CT chest changes − SNHL 11.8 years
22	6	M	gp91	− Recurrent lymphadenitis − Prolonged diarrhea − Failure to thrive	MUD	Camp Bu Cy	No	No	​	− Eczema (T)	None 13.3 years
23	5	M	gp91	− Recurrent chest infections − Mouth ulcers − Anal fistula − Pericardial effusion	MSD	Bu Cy	No	No	​	− Hyper-transaminitis (T) − Chronic abdominal pain (P) − Pectus excavatum (P) − Gum inflammation (P) − Acne (P)	− Chronic abdominal pain (P) − Pectus excavatum (P) − Gum inflammation (P) − Acne (P) 13.2 years
24	2	M	gp91	− BCG adenitis − Perianal abscesses	MSD	ATG Bu Flu	No	No	​	− Osteochondroma (P) − Advanced bone age for pubertal age (P)	− Osteochondroma − Advanced bone age for pubertal age 10.3 years
25	7	M	gp91	− PVL *S. aureus* skin abscesses − Liver abscess − Lymphadenitis − Fungal chest infections − Balanitis	MUD	Camp Flu Bu	No	No	DLI	− Underweight (T) − Conjunctivitis (T) − Recurrent colitis (I)	− Recurrent colitis 11.3 years
26	12	M	gp91	− Positive family history − Liver abscess − Recurrent URTI	MSD	Bu Cy	No	No	​	− Skin VZV (T) − Aspermia (P)	− Aspermia 3.5 years
27	6	F	p67	− Recurrent LRTI − Failure to thrive	MSD	ATG Bu Flu	No	No	​	− Difficulties at school (pre and post) (P) − Recurrent mild ear infections (P) − Conductive hearing loss (pre and post) (P) − Gum pain (P)	− Difficulties at school − Recurrent mild ear infections − Conductive hearing loss − Gum pain 8.6 years
28	14	M	gp91	− Fungal driven granulomatous mediastinal mass causing SVC obstruction	MUD	Camp Flu Bu	Grade I skin	Joint involvement	​	− Delayed puberty (T) − GH deficiency (T) − Anxiety with needle phobia (T) − Bilateral lower lid lash misdirection and severe blepharitis (T) − Voriconazole photosensitivity (T) − Hypothyroidism (P) − SNHL (P) − Bowen’s disease (P) − Squamous cell carcinoma (P)	− Hypothyroidism − SNHL − Bowen’s disease − Squamous cell carcinoma 5 years
29	12	M	gp91	− CGD colitis − Perianal disease − Lymphadenitis − Lymph nodes granulomatous lesions	MUD	Camp Flu Bu	Grade I skin	No	​	− Dyslipidemia (T) − Tremors (T) − Keratosis pilaris (T) − Autoimmune hyperthyroidism (P) − Overweight (P) − Sleep difficulties	− Hay fever and blocked nose − Autoimmune hyperthyroidism Overweight − Sleep difficulties 9.2 years
30	11	M	gp91	− Perianal abscesses − Recurrent LRTI	MMUD	Camp Flu Bu	No	No	​	− Adrenal suppression (T) − Delayed puberty (T) − Short stature (T) − Residual CT chest changes (P) − Axonal neuropathy (P) − Anxiety (P)	− Residual CT chest changes − Axonal neuropathy − Anxiety 8.3 years
31	2	M	gp91	− Neonatal pustular rash − Recurrent chest infections − Transient diarrhea − Gastric outlet obstruction − Short stature	MMUD	Camp Flu Bu	Grade II skin	No	​	− Low body density (T) − Overweight (P) − Osteochondroma (P) − Spindle cell sarcoma of the neck (P)	− Overweight − Osteochondroma − Spindle cell sarcoma of the neck 7.4 years
32	9	M	p47	− Positive family history	MUD	Camp Flu Treo	Grade II skin	No	​	− Recurrent arthralgia (T) − Eczema (T) − Overweight/Obesity (P) − Behavioral difficulties (P)	− Overweight − Behavioral difficulties 6.1 years
33	3	M	gp91	− Staph aureus and Pseudomonas skin infections − Groin abscess	MSD	ATG Bu Flu	No	No	​	− Leg pain (T) − School difficulties (P) − Dry skin (P)	− School difficulties − Dry skin 7 years
34	8	M	p47	− CGD colitis	MUD	Camp Flu Treo	Grade II skin	No	​	− Chicken pox (T) − Joint stiffness (T) − Chronic EBV viremia (P) − Adrenal insufficiency (P) − Delayed puberty (P) − Gynecomastia (P) − Sleep difficulties (P)	− Chronic EBV viremia − Adrenal insufficiency − Delayed puberty − Gynecomastia − Sleep difficulties 6.4 years
35	6	M	p67	− Crohn’s like IBD orofacial granulomatosis − Lymphadenitis	MSD	ATG Bu Flu	No	No	​	− Overweight (T)	None 5.9 years
36	12	M	p47	− Staph aureus liver abscess − Aspergillus lung infection	MUD	Camp Flu Treo	Grade I skin	No	​	− PRES and increased ICP (T) − Adrenal insufficiency (P) − Obesity (P)	− Adrenal insufficiency − Obesity 4.9 years
37	1	M	gp91	− Fungal chest infection	MMUD	ATG Flu Treo Thio	Grade III gut	No	​	− Short stature (T) − Adrenal insufficiency (P) − Obesity/Overweight (P) − Low bone density (P) − Neurodevelopmental delay (pre and post) (P)	− Adrenal insufficiency − Overweight − Low bone density 4.6 years
38	5	F	p47	− Family history − Skin granulomas − Angular cheilitis, Pseudomonas infection − *Rothia* bacteremia − Ground glass change	MUD	Camp Flu Treo	No	No	​	− ITP (T) − Borderline hearing threshold (T) − Adrenal insufficiency (P)	− Adrenal insufficiency 5.2 years
39	5	M	gp91	− Family history − Skin granuloma	MUD	Camp Flu Treo	Grade I skin	No	​	− Chronic EBV viremia (T)	None 5.2 years
40	6	M	gp91	− *Serratia* lymphadenitis − Anal abscesses − Perianal opening fistula	MUD	Camp Flu Treo	Grade I skin	Skin, mouth	DLI	− VZV reactivation (T) − Adrenal insufficiency (P) − Short stature (P) − Anxiety (P)	− Adrenal insufficiency − Short stature − Anxiety 4.9 years
41	13	F	p22	− β thalassemia trait − Crohn’s colitis − Liver abscess − Intra-abdominal and thoracic lymphadenopathy − Atypical mycobacteria infection	MUD	Camp Flu Treo	No	No	DLI	− Bloody diarrhea (at last visit) − Residual liver hypertrophy (P) − Hyper-transaminitis (P) − Thyroiditis (P) − Underweight (P) − Low bone density (P) − Anxiety (P) − SNHL (P) − Dental malalignment (P)	− Bloody diarrhea − Residual liver hypertrophy − Hyper-transaminitis − Thyroiditis − Underweight − Low bone density − Anxiety − SNHL − Dental malalignment 4.9 years
42	11	M	gp91	− Recurrent otitis media − Perianal abscess − Colitis − Angular cheilitis − Diffused ground glass lung opacification and fibrosis process	MMUD	ATG Flu Treo Thio Rituximab	Grade I gut	No	​	− Residual CT chest changes (P) − Adrenal insufficiency (P) − Delayed puberty (P)	− Residual CT chest changes − Adrenal insufficiency − Delayed puberty 2.4 years

ATG, anti-thymocyte globulin; BCG, bacillus Calmette-Guerin; Bu, busulfan; Camp, Campath (alemtuzumab); CNS, central nervous system; Cy, cyclophosphamide; EBV, Epstein–Barr virus; Flu, fludarabine; GH, growth hormone; HSV, herpes simplex virus; HTN, hypertension; ICP, intracranial pressure; ITP, immune thrombocytopenia; LRTI, lower respiratory tract infection; Melph, melphalan; NEC, necrotizing enterocolitis; NTM, nontuberculous mycobacteria; OCD, obsessive-compulsive disorder; OMA, acute otitis media; PRES, posterior reversible encephalopathy syndrome; PVL, periventricular leukomalacia; SNHL, sensorineural hearing loss; SVC, superior vena cava; TG, thyroglobulin; Thio, thiotepa; Treo, treosulfan; URTI, upper respiratory tract infection; VZV, varicella zoster virus; MFD, matched family donor.

### Pretransplant comorbidities

Inflammatory complications were frequent, with gastrointestinal involvement most prominent. 17 patients (40.5%) presented with CGD-related colitis, including three cases of fistulizing disease. Two additional patients (4.8%) exhibited perianal fistulae, while three patients (7.1%) presented with non-bloody diarrhea, all without diagnostic confirmation of IBD (no endoscopy or fecal calprotectin assessment performed/available from records). Moreover, six patients (14.3%) presented with perianal abscesses.

Pulmonary manifestations were observed in 20 patients (47.6%), including bacterial and fungal chest infections, granulomatous lung disease, interstitial changes on chest imaging, and extrinsic allergic alveolitis.

Other significant organ involvement included liver abscesses being recorded in eight patients (19.0%), severe cerebral fungal infections in two patients (4.8%), pericardial effusion in two patients (P11 and P23 [18]), one case with recurrent osteomyelitis (P12), and another with recurrent pancreatitis in the context of interferon-γ (IFN-γ) therapy for *Mycobacteria chelonae* sepsis (P17). P9 presented with recurrent fevers without any localized organ involvement.

### Transplant characteristics

A total of 45 transplant procedures were performed in 42 patients, with three patients requiring a second HSCT. The comprehensive characteristics of patients and transplant procedures are detailed in Table 1. Regarding donor selection, 10/10 matched unrelated donors (MUDs) were utilized in 23 procedures (51.1%), matched sibling donors (MSDs) in 11 procedures (24.4%), and one patient (2.2%) received cells from matched family donor. The remaining 10 patients (22.2%) underwent transplantation from 9/10 human leukocyte antigen-mismatched unrelated donors (MMUDs).

Regarding stem cell source, bone marrow (BM) was used in 25 transplants (55.5%), peripheral blood stem cells (PBSCs) in 18 (40.0%), and cord blood in 2 (4.4%).

Conditioning regimens were predominantly busulfan based, employed in 30 of 45 procedures (66.7%), with 11 transplants following a myeloablative conditioning (MAC). Treosulfan-based conditioning was administered in 14 procedures (31.1%, treosulfan/fludarabine; *n* = 12 and treosulfan/fludarabine/thiotepa; *n* = 2), while one patient received melphalan-based conditioning.

Acute graft-versus-host disease (aGvHD) occurred in 25/45 procedures (22 after first HSCTs and three after second transplants), involving 24 patients; one patient developed aGvHD after both procedures. Among these, grade I was observed in 13 patients, grade II in nine patients, and grade III in two patients. Additionally, P17 developed pericardial effusion as a manifestation of aGvHD. Chronic GvHD (cGvHD) was observed in six cases (6/42, two cases after a second transplant; 14.3%), with three presenting as limited skin cGvHD and three as extensive cGvHD with variable involvement of gastrointestinal tract, joint involvement, and ocular manifestations (Table 2).

### Survival outcomes

#### Overall survival (OS)

**Figure 1.**
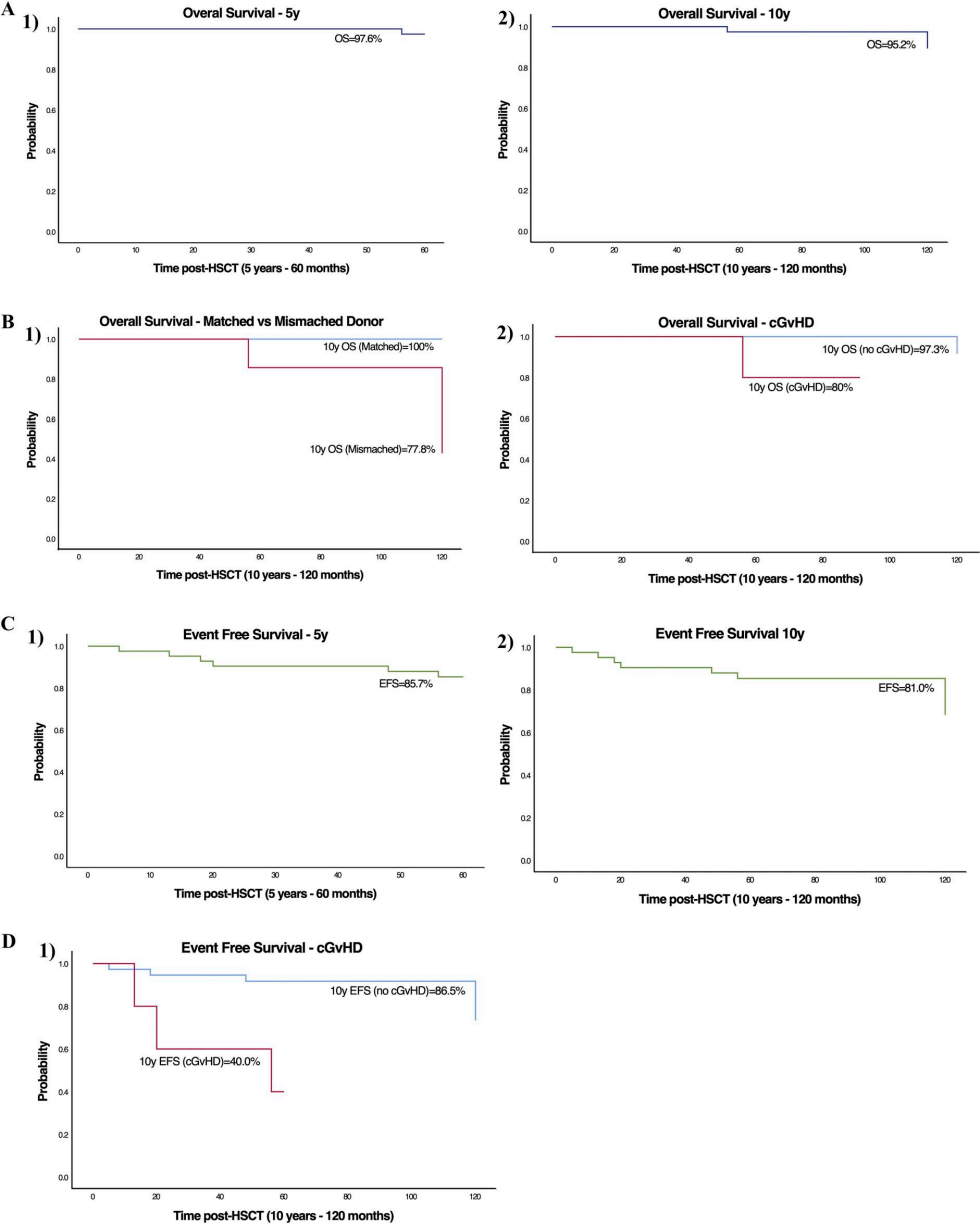
**OS and EFS. (A)** OS at 5–10 years (y) after transplant for our cohort (*n* = 42). **(B)** Impact on 10-year OS of matched or mismatched donor (P = 0.002) and of cGvHD (P = 0.001). **(C)** EFS at 5–10 years after transplant for our cohort (*n* = 42). **(D)** Impact on 10-year EFS of cGvHD (P = 0.001).

Among the studied cohort, the 5- and 10-year OS rates were 97.6% and 95.2%, respectively (Fig. 1, A1–A2). Two patients had late death (>2 years after HSCT). P9 received a 9/10 MMUD HSCT (reduced intensity conditioning [RIC], BM) and developed BK polyomavirus (BK) nephropathy that led to progressive renal failure and death 4 years after HSCT; donor chimerism was 95% in whole blood at time of death. P18 received a 9/10 MMUD HSCT (RIC, PBSC) and maintained full donor engraftment until the transition to adult care. 10 years after HSCT, at 25 years old, he suffered a sudden, unexplained cardiac arrest and passed away (19).

10-year OS was better in fully matched HSCT recipients compared to MMUD recipients (Fig. 1 B1, P *= *0.002). The presence of cGvHD was associated with lower OS (Fig. 1 B2, P *= *0.001).

#### Event-free survival (EFS)

Among the studied cohort, the 5- and 10-year EFS rates were 85.7% and 81.0%, respectively (Fig. 1, C1–C2). The presence of cGvHD was associated with reduced 10-year EFS*,* P *= *0.001 (Fig. 1 D1). Reduced donor myeloid chimerism correlated with an increase in adverse events, with 0% EFS in those with low donor myeloid chimerism (<25%), 80% EFS in mixed chimerism (25–94%), compared with 93% EFS in full donor chimerism (≥95%); P *= *0.001. Data on myeloid engraftment were missing for three patients.

Kaplan–Meier analysis showed that 10-year OS and 10-year EFS were not significantly influenced by the age at HSCT (≤5 vs. >5 years), CGD diagnosis (XL vs. AR), presence of colitis, lung disease, liver abscesses, or perianal disease, stem cell source (BM, PBSC, and cord blood), conditioning regimen (RIC vs. MAC), use of serotherapy, occurrence of aGvHD, presence of autoimmunity within the first 2 years after HSCT, and early immune recovery markers (CD4^+^ ≥300 vs. <300 at 6 mo, CD4^+^ ≥500 vs. <500 at 6 mo, and CD3^+^ ≥1,000 vs. <1,000 at 6 mo after HSCT). Full details are provided in Table S2.

### Second intervention

Six patients (P10, P12, P21, P25, P40, and P41; 14.3%) required a second therapeutic intervention following their initial transplant. Three patients received donor lymphocyte infusion (DLI) alone; two underwent DLI, followed by a second HSCT. P21 received gene therapy on a compassionate basis (SFgp91phox gammaretroviral vector with melphalan conditioning) followed by a second HSCT. Detailed characteristics of these cases, including CGD subtype, transplant characteristics, CGD-related complications pre- and post-intervention, chimerism status, and clinical outcomes, are presented in Table 3.

**Table 3.** Description of patients needed second interventions, including genetic background, comorbidities, second intervention characteristics, and outcomes

​	P10		P12		P21		P25	P40	P41
CGD	gp91		p67		gp91		gp91	gp91	p22
Comorbidities	Colitis Perianal disease Lung involvement		Colitis		Lung involvement		Lung involvement Liver abscess	Colitis Perianal disease	Lung involvement Colitis Perianal disease
Age at first HSCT (years)	11.4		3.2		4.4		7.2	6.4	13.2
Donor	MUD		MUD		MUD		MSD	MUD	MUD
Source	BM		PBSC		PBSC		BM	PBSC	PBSC
Conditioning	Camp/Bu/Cy (MAC)		Camp/Flu/Treo (RIC)		Camp/Flu/Melph (RIC)		Bu/Cy (RIC)	Camp/Flu/Treo (RIC)	Camp/Flu/Treo (RIC)
aGvHD/cGvHD	No/Yes		No/No		Yes/Yes		No/No	Yes/yes	No/No
Type of second intervention	DLI (1 × 10^7^/kg)	Second HSCT	DLI (1 × 10^6^/kg)	Second HSCT	Gene therapy	Second HSCT	DLI (1, 5, 10 × 10^7^/kg)	DLI (1 × 10^7^/kg)	DLI (1, 5 × 10^7^/kg)
Time from first HSCT to second intervention (years)	1.7	3.3	0.3	1.6	1.3	3.9	9.8	1	1.4
Cause of second intervention	Declined in donor chimerism	Declined in donor chimerism	Declined in donor chimerism	Declined in donor chimerism	Secondary graft failure	Persistent graft failure post gene therapy	Declined in donor chimerism	Declined in donor chimerism	Declined in donor chimerism
CGD-related disease before second intervention	Relapse of colitis	Relapse of colitis	None	None	De novo colitis Recurrence of lung involvement	None	De novo perianal fistulizing disease	No	No
Chimerism at second intervention	WB 19%	WB 11%	CD3 61% CD15 29%	CD3 58% CD15 0%	WB 0%	WB 0%	CD3 48% CD15 23%	CD3 59% CD15 19%	CD3 57% CD15 11%
Age at second intervention (years)	13.1	14.8	3.5	4.7	5.7	8.3	17	7.4	14.6
If second HSCT, specify donor-source-conditioning-aGvHD/cGvHD	MMUD-PBSC- Camp/Flu/Treo (RIC) No/Yes		MUD-PBSC-Camp/Flu/Bu (MAC) Yes (stage-II skin)/No		MUD-PBSC-Camp/Bu/Cy (RIC) Yes/Yes (eyes and skin-limited sclerodermatous)		/	/	/
CGD-related disease after second intervention	None		None		None		Fistulizing disease	None	None
On IS at last FU	No		No		No		No	No	No
Chimerism at last FU	WB 100%, CD15 100%		CD3 100%, CD15 100%		CD3 100%, CD15 100%		CD3 51%, CD15 100%	CD3 100%, CD15 100%	CD3 59%, CD15 10%; patient declined second transplant

Bu, busulfan; Camp, Campath; Cy, cyclophosphamide; Flu, fludarabine; FU, follow-up; Melph, melphalan; Treo, treosulfan.

The primary indications for a second intervention were mixed chimerism (*n* = 5) and graft failure (*n* = 1). In this subgroup, the median age at initial HSCT was 6.8 years (range 3.2–13.2 years), with a median interval of 1.7 years (range 0.3–9.8 years) before requiring second intervention.

All patients initially received HSCT from either MUD or MSD. PBSCs were the predominant stem cell source (4/6, 66.7%), and RIC was employed in most cases (5/6, 83.3%).

All patients initially achieved myeloid engraftment levels exceeding 95%. Five cases (P10, P12, P25, P40, and P41) had a progressive decline in myeloid donor chimerism observed in the first year after HSCT.

Of note, P25 maintained myeloid donor chimerism >60% for 6 years after HSCT. He was lost to follow-up for a 2-year period, and subsequently, at 9 years after HSCT, he presented with perianal fistulizing disease (biopsies of the fistula track revealed granulomatous inflammation suggestive of Crohn’s disease), requiring colostomy. Myeloid chimerism dropped to 42% with nitroblue tetrazolium test (NBT) of 12%. He received three escalating doses of DLI (1 × 10^7^/kg, 5 × 10^7^/kg, and 1 × 10^8^/kg) with reversion to donor myeloid engraftment of 100% at 1 year following the first DLI and remained stable at time of transition (2 years and 2 mo following first DLI).

All patients apart from P41 went for a second or third intervention until stabilization of donor chimerism. P41 received a MUD transplant (PBSC and RIC), demonstrated initial 100% donor chimerism, followed by slipping myeloid engraftment from 100 day after HSCT reaching CD15^+^ myeloid chimerism of 11%. Despite receiving two increasing doses of DLI (1 × 10^7^/kg; 5 × 10^7^/kg), no improvement in myeloid engraftment was observed. At transition to adult services, the patient was 18 years old, demonstrated no evidence of CGD-related disease, maintained donor myeloid chimerism of 10%, and showed nitroblue tetrazolium test results of 19%. The patient declined a second HSCT.

### Chimerism and immune reconstitution

**Figure 2.**
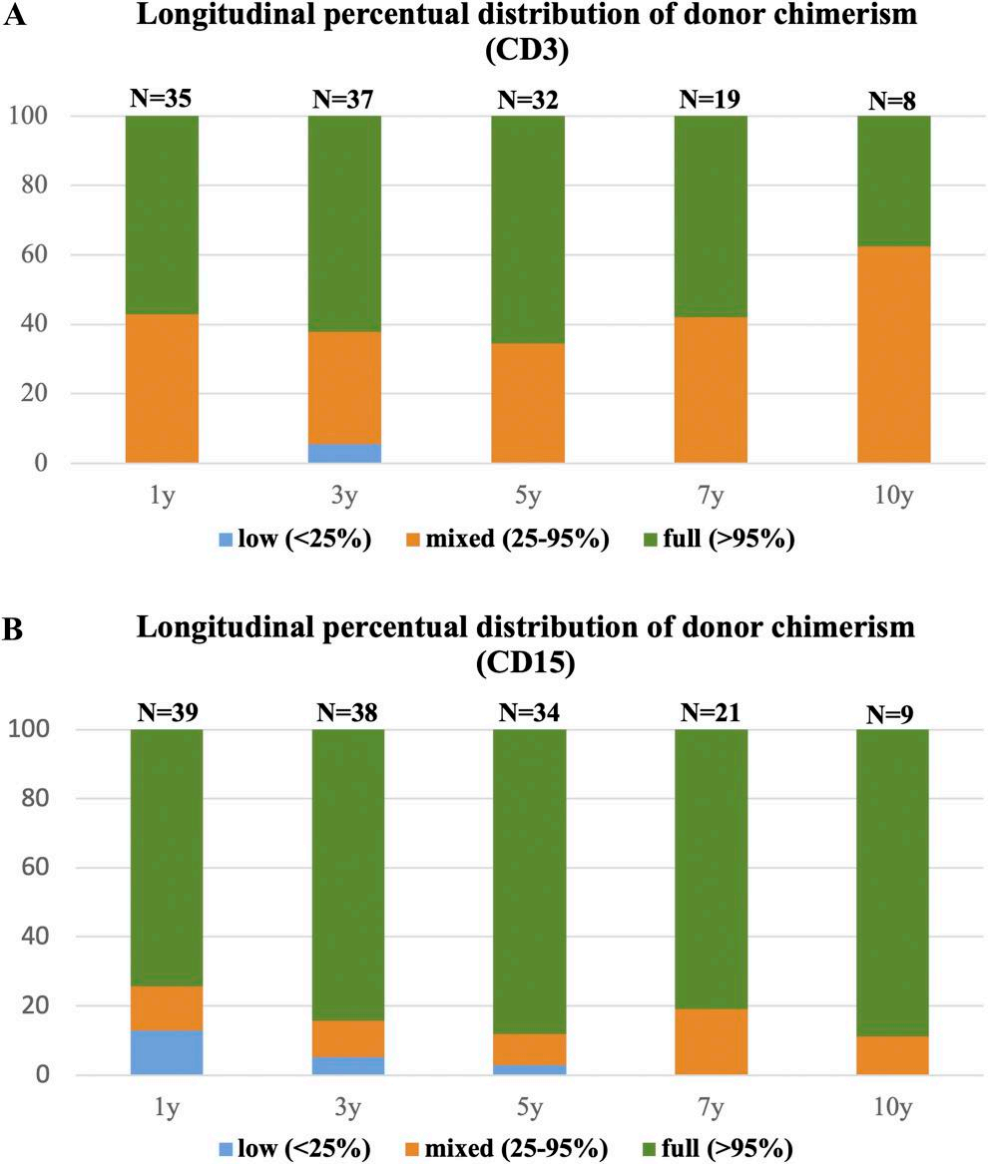
**Posttransplant peripheral blood chimerism (*n* = 42). (A)** Longitudinal distribution of CD3^+^ blood chimerism at 1, 3, 5, 7, and 10 years after transplant. **(B)** Longitudinal distribution of CD15^+^ blood chimerism at 1, 3, 5, 7, and 10 years after transplant.

Chimerism analysis at final follow-up was available for 41 of 42 patients, with a median posttransplant assessment time of 6.5 years (range 1.2–13.6 years). Durable full donor chimerism in peripheral blood (myeloid and/or whole blood chimerism ≥95%) was achieved in 37 of 41 patients (90.2%). Three patients (7.3%) demonstrated mixed chimerism (25–95%), while one patient (P41) maintained very low-level myeloid engraftment (<25%). The longitudinal distribution of lineage-specific chimerism, including myeloid (CD15^+^) and T lymphocyte (CD3^+^) fractions, evaluated at predetermined time points (1, 3, 5, 7, and 10 years after transplant) is presented in Fig. 2.

The median time from the first procedure to achieve CD4^+^ counts ≥300 cells/μl was 10.3 and 14.4 mo for CD3^+^ counts ≥1,000 cells/μl.

Posttransplantation vaccine responses were available in 29 cases: among these, 25 patients (86.2%) developed protective antibody titers against tetanus toxoid, while 22 (75.9%) demonstrated seroprotection against at least 7 of 13 pneumococcal serotypes tested.

Two patients were still on immunosuppressive therapy at last follow-up (P8 and P9), with P9 being immunoglobulin-dependent at the time of death.

### Long-term complications

Detailed patient characteristics, clinical presentations, transplant parameters, and posttransplant complications are presented in Table 2.

### Autoimmune complications

Posttransplant autoimmune complications occurred in 14 of 42 patients (33.3%), with detailed characteristics summarized in Table 4. The majority had hematological autoimmunity (7/14; 50%), with Coombs’ positive autoimmune hemolytic anemia (AIHA) occurred in five patients (P8, P17, P31, P37, and P38) and autoimmune thrombocytopenia (AIT) with platelet antibodies occurred in two patients (P20 and P36). Other autoimmune manifestations included thyroid disorders such as Graves’ disease (P29), Hashimoto’s thyroiditis (P7), euthyroid autoimmune thyroiditis, (P41), autoimmune polyarthritis (P21), and neurological complications (acute disseminated encephalomyelitis, ADEM, P15; or Guillain-Barré-like syndrome, GBS-like, P12 and P30).

**Table 4.** Description of patients with autoimmunity after transplant

​	P7	P8	P12		P15	P17	P20	P21		P29	P30	P31	P36	P37	P38	P41
CGD	gp91	gp91	p67		gp91	gp91	p47	gp91		gp91	gp91	gp91	p47	gp91	p47	p22
Clinical presentation at diagnosis	Liver and skin abscess CGD colitis Extrinsic allergic alveolitis	Invasive Aspergillosis BCG-osis	CGD colitis Osteomyelitis		CGD colitis Urethral granuloma	Lymphadenitis Pancreatitis Liver abscess Pneumonia Sepsis ITP	Skin and perianal abscesses Recurrent URTI/OMA Asthma	Recurrent LRTI		Colitis Perianal disease Lymphadenitis	Perianal disease Recurrent LRTI	Neonatal rash Recurrent LRTI Diarrhea Gastric obstruction Short stature	Liver abscess Fungal chest infection	Fungal chest infection	Skin granulomas Pseudomonas infection *Rothia* bacteremia Ground glass change	Colitis Liver abscess Lymphadenopathy Atypical mycobacteria infection
Age at HSCT (years)	3.4	0.9	3.1 (first)	4.4 (second)	5.4	6.3	8.7	4.4 (first)	8.2 (second)	12.4	10.6	2.2	12.3	1.5	5.4	13.2
Donor	MMUD	MUD	MUD	MMUD	MSD	MMUD	MUD	MUD	MUD	MUD	MMUD	MMUD	MSD	MUD	MMUD	MUD
Source	BM	PBSC	PBSC	PBSC	BM	Cord	BM	PBSC	PBSC	BM	BM	BM	BM	Cord	PBSC	PBSC
Conditioning	RIC	RIC	RIC	RIC	MAC	MAC	RIC	RIC	RIC	RIC	RIC	RIC	RIC	MAC	RIC	RIC
aGvHD	Yes	Yes	No	Yes	No	Yes	Yes	No	Yes	Yes	Yes	Yes	Yes	Yes	No	No
cGvHD	No	No	No	No	No	No	No	No	Yes	No	No	No	No	No	No	No
Autoimmunity1 (AI1)	Hashimoto	AHIA	GBS-like		ADEM	AIHA	AIT	Polyarthritis		Grave’s disease	GBS-like	AIHA	AIT	AIHA	AIHA	Thyroid swelling with +ab
Time to AI1 (m)π	80	6	28		14	13	9	51		58	4	4	3	14	8	49
Chimerism at AI1 onset	WB 100%	WB 100%	WB 100%		WB 100%	WB 100%	WB 100%	WB 100%		CD3 53%, CD15 100%	WB 98%	CD3 89%, CD15 93%	WB 100%	WB 100%	CD3 63%, CD15 100%	CD3 67%, CD15 9%
Treatment for AI1	Levothyroxine	Steroids-MMF-Rituximab-Sirolimus	Steroids-IVIG-Gabapentin		Steroids	Steroids-Rituximab	IVIG	Steroids		Propranolol-Carbimazole-Levothyroxine	Steroids-NCA-Acetazolamide-Amitriptyline-Gabapentin	Rituximab	IVIG-Steroids-Rituximab	Steroids-Rituximab	Steroids-Rituximab	None
Autoimmunity2 (AI2)	​	​	​		​	Immune complex glomerulonephritis	​	​		​	​	Nephrotic syndrome	Autoimmune neutropenia	​	ITP	/
Chimerism at AI2 onset	​	​	​		​	CD3 86%, CD15 100%	​	​		​	​	CD3 89%, CD15 93%	CD3 100%, CD15 100%	​	CD3 64%, CD15 79%	​
Time to AI2 (mo) π	​	​	​		​	97	​	​		​	​	5	3.7	​	29	​
Treatment for AI2	​	​	​		​	Steroids-MMF-Tacrolimus-Enalapril	​	​		​	​	​	​	​	​	​
Any AI in the first 2 years	No	Yes	No		Yes	Yes	Yes	No		No	Yes	Yes	Yes	Yes	Yes	No
Any AI >2 year	Yes	Yes	Yes		No	Yes	No	Yes		Yes	No	No	No	No	Yes	Yes
Second intervention	None	None	DLI + second HSCT		None	None	None	Gene therapy + second HSCT		None	None	None	None	None	None	DLI
IS at last FU	None	Sirolimus-Steroid	None		None	None	None	None		None	None	None	None	None	None	None
Outcome AI1-AI2	Stable on treatment	Persisting	Resolved		Resolved	Resolved, stable on treatments	Resolved	Resolved		Stable on treatments	Resolved	Resolved	Resolved	Resolved	Resolved	Monitoring

AI1, first autoimmunity episode; AI2, second autoimmunity episode; BCG, bacillus Calmette-Guerin; IS, on immunosuppressives; IVIG, intravenous immunoglobulin; LRTI, lower respiratory tract infection; MMF, mycophenolate mofetil; NCA, neurocirculatory asthenia; OMA, otitis media acute; PBSC, peripheral blood stem cell; URTI, upper respiratory tract infection. π: time to AI1 or time to AI2 is time from first HSCT to development of AI1 or AI2.

The median time to the first autoimmune manifestation was 13 mo after HSCT (range 3–80 mo), with most of the cases (64.3%) developing autoimmunity within the first 2 years after HSCT. Eight patients developed relapsing or late-onset (>2 years) autoimmune conditions. Multiple autoimmune episodes were observed in four patients (P17, P31, P36, and P38). Of note, among AIHA cases, three patients developed secondary autoimmune conditions (immunocomplex glomerulonephritis, nephrotic syndrome, and AIT at a median of 17 mo after transplant, range 3.7–97 mo).

Notably, three patients (3/14; 21%) developed autoimmune complications following a secondary intervention: GBS-like presentation (27.5 mo after initial HSCT and 8 mo after second transplant), autoimmune polyarthritis (50.7 mo after initial HSCT and 3 mo following second transplant), and autoimmune thyroiditis manifesting as hyperthyroidism (48.6 mo after initial HSCT and 30.8 mo after DLI).

No correlation was identified between autoimmunity and chimerism levels—both as myeloid and T cell donor engraftment—at time of autoimmunity. At autoimmune diagnosis, full donor T cell chimerism was present in 10 patients (71.4%), while four showed mixed T cell chimerism (all had donor T cell engraftment >50%). 12 patients (86.0%) demonstrated full myeloid chimerism, one had mixed CD15^+^ chimerism (P31, myeloid donor engraftment of 30%), and one showed very low CD15^+^ chimerism (P41; myeloid donor engraftment of 9%).

Regarding long-term management, only P8 remained on immunosuppressives for relapsing AIHA at last follow-up at 9 years of age. P7 and P29 with thyroid disorders (Hashimoto’s and Graves’ disease) remained stable on specific therapy, while P41 with autoimmune thyroiditis and thyroid swelling maintained euthyroid status and is currently under surveillance. The patient with immunocomplex glomerulonephritis achieved clinical stability on angiotensin-converting enzyme inhibitors and was able to discontinue all immunosuppressive medications.

### Organ-specific long-term complications

**Figure 3.**
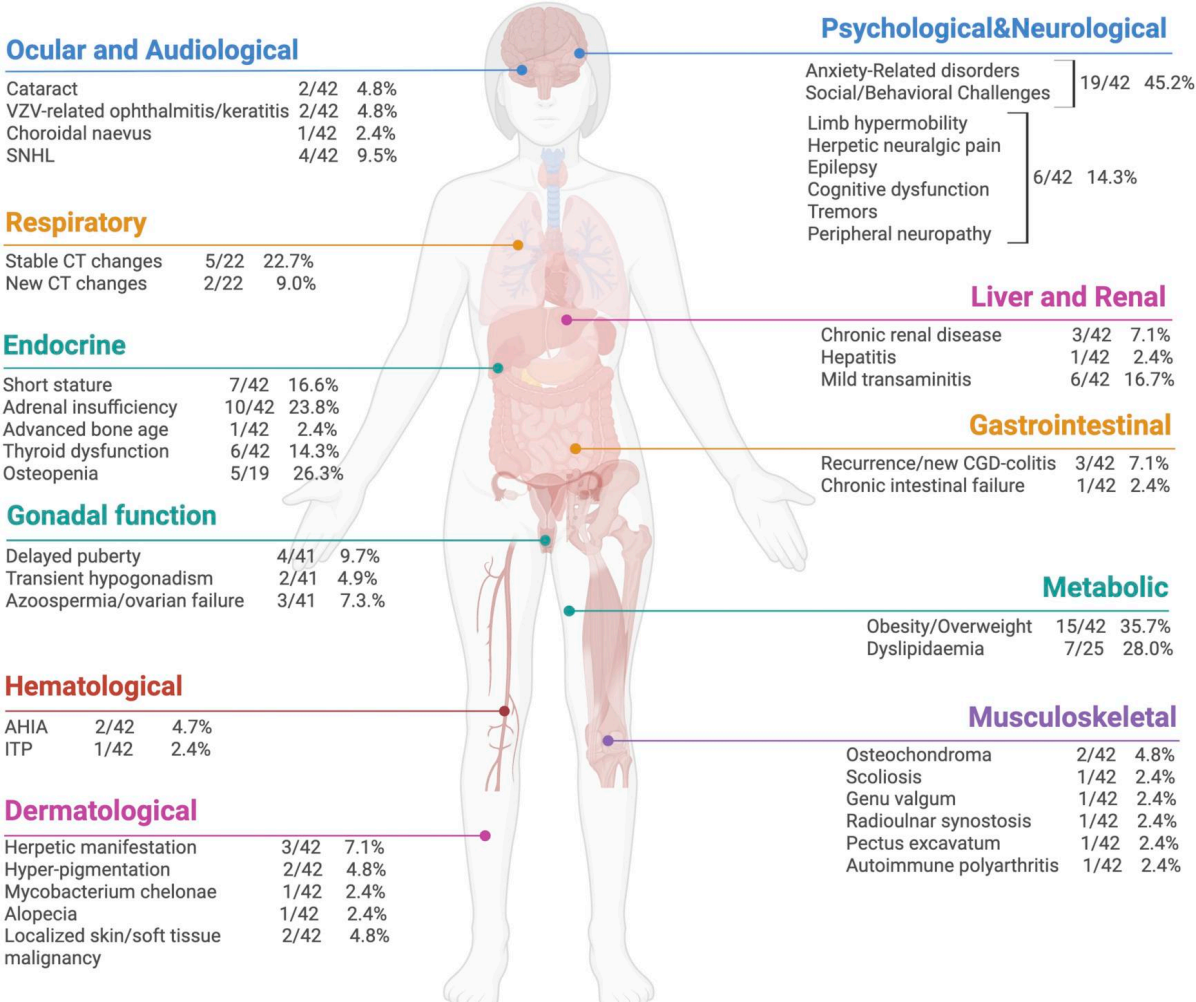
Long-term specific complications (made by BioRender).

Long term specific complications are detailed in Fig. 3.

#### Pulmonary outcome

Pulmonary function testing was performed in 33 patients, with 23 patients (70%) demonstrating normal function at final follow-up. Among the 10 patients with abnormal results, three exhibited specific abnormalities, including mild airway obstruction and impaired gas exchange; two of these patients had documented pretransplant lung involvement. Seven patients were unable to complete full pulmonary function testing due to age limitations. Among them, two had normal spirometry but were unable to undergo diffusing capacity of the lung for carbon monoxide (DLCO) assessment.

Computed tomography (CT) was performed in 22 patients. 15 patients (68.2%) showed either normal findings or regression of previous lung pathology. Five cases showed evidence of stable CT findings in comparison to pre-HSCT, while two had radiological evidence of progressive lung disease (traction bronchiectasis in P21 and nodular consolidation in P16).

#### Gastrointestinal outcome

Of 17 patients with pretransplant colitis, 16 (94%) achieved complete resolution posttransplantation apart from P10 who developed relapsing colitis concurrent with declining chimerism, which resolved following second intervention.

Nevertheless, two patients developed new-onset gastrointestinal complications coinciding with decline in whole blood engraftment to 0% in P21 and myeloid engraftment of 42% in P25. P21 presented with de novo colitis, while P25 developed de novo fistulizing perianal disease. Both conditions showed improvement following DLI intervention.

In addition, P9 developed chronic intestinal failure associated with immune reconstitution (GvHD) and remained dependent on parenteral nutrition until time of death.

#### Cardiac outcome

24 patients had a formal cardiac assessment with two (2/34; 5.9%) having abnormalities. P7 had isolated premature ventricular contractions. P37 was noted to have transient left ventricular hypertrophy and aortic root dilation 2 years after transplantation, attributed to steroid therapy, which resolved by 3 years after transplantation.

#### Hepatic outcome

Seven patients (16.6%) developed transaminitis at a median of 5.1 years after transplantation (range 2.2–15.2 years). One case was attributed to herbal medicine-induced hepatitis, which resolved spontaneously. Of the remaining six patients, three demonstrated persistent elevated alanine/aspartate aminotransferase (AST) levels at final follow-up (P6, P9, and P41), while one patient showed persistent mild elevation in gamma-glutamyl transferase (GGT) (P11).

#### Renal outcome

Three patients (7.1%) developed late-onset renal disease at a median of 2.4 years after HSCT (range 0.4–8.2 years). The first patient (P9) progressed to stage IV chronic kidney disease 2.3 years after transplantation following BK virus-associated nephropathy. The second patient (P17) developed biopsy-confirmed immunocomplex glomerulonephritis 8 years after transplant, resulting in persistent stage III chronic kidney disease. The third patient developed nephrotic syndrome while receiving cyclosporine and prednisolone for AIHA, which resolved with high-dose prednisolone therapy (P31).

### Late endocrine effects

#### Metabolic outcome

At a median of 5 years after transplantation (range 0.16–8.8 years), 15 of 42 patients (35.7%) met criteria for either overweight (body mass index [BMI] >91st centile) or obesity (BMI >98th centile). At last follow-up, 11 patients (26.2%) remained either overweight (*n* = 8) or obese (*n* = 3).

Hypertension was documented in three patients (7.1%), with two cases associated with underlying renal disease. Among 25 patients evaluated for dyslipidemia, seven demonstrated hypertriglyceridemia, with five showing persistent abnormality at last follow-up (two of them with concomitant persistent overweight status at last follow-up).

#### Bone health outcome

Dual-energy X-ray absorptiometry (DEXA) scanning was performed in 19 patients at a median of 3.2 years after transplantation, with five patients (26.3%) showing evidence of osteopenia. Vitamin D levels were assessed in 26 patients, with deficiency documented in five cases.

Fractures were recorded in two cases (P8 and P14). P8 had a tibial fracture 4 years after HSCT following prolonged steroid therapy for relapsing AIHA; notably, this patient had not undergone a DEXA scanning. P14 experienced jaw and elbow fractures 3 years after HSCT, which were associated with low bone mineral density at DEXA scan. Following the fractures, he was started on calcium supplementation and regular monitoring.

Non-osteopenic skeletal complications were identified in six patients (14.3%) at a median of 7.7 years after transplantation (range 2.8–11.9 years), including genu valgum (P6), radioulnar synostosis (P11), scoliosis (P18), pectus excavatum (P23), and osteochondromas (P24 and P31).

Within our cohort of patients, we did not observe an association between conditioning type (busulfan versus treosulfan), aGvHD (any versus none), post-HSCT autoimmunity (presence versus absence), use of systemic steroids (yes versus none), and any non-osteopenic bone pathology (details of the analysis are included in the supplementary document Table S3).

#### Gonadal outcome

Gonadal function and comprehensive endocrine evaluation were performed in 41 of 42 patients. While 32 patients demonstrated normal hormonal profiles and clinical assessments, nine patients showed abnormalities (22.0%).

Among girls, two patients (P11, P20) developed secondary ovarian failure requiring hormonal replacement therapy (HRT), while one patient (P3) had delayed puberty successfully achieved following HRT. Among boys, transient hypogonadism was noted in two patients (P28, P15), delayed puberty in three cases (P34, P42, P30). Of note, P30 achieved puberty with HRT help and others are awaiting therapy. Sperm analysis is not part of the assessment in our pediatric/adolescent center; however, P26, who went for sperm collection, was noted to have confirmed azoospermia.

Within our cohort of patients, we did not observe an association between conditioning type (busulfan versus treosulfan), conditioning intensity (MAC versus RIC), or gender (male versus female) and any gonadal outcome. Given the sample size, results need to be interpreted with caution (details of the analysis are included in the supplementary document, Table S4).

#### Growth outcome

Short stature was observed in seven patients (16.6%) at a median of 2.7 years after transplantation (range 1.5–11.4 years). Contributing factors included adrenal insufficiency (*n* = 4) and growth hormone deficiency (*n* = 2), with one patient requiring ongoing replacement therapy due to empty sella syndrome. One patient with advanced bone age commenced anastrozole therapy to optimize final height potential. At final assessment, three patients continued to have short stature (P8, P9, and P40) at last follow-up.

#### Additional endocrine complications

Thyroid dysfunction was documented in six patients (14.3%): transient thyroid-stimulating hormone (TSH) elevation (P4), non-autoimmune hypothyroidism (P11, P28), autoimmune hyperthyroidism (P29) on antithyroid therapy plus symptomatic β-blockers, autoimmune hypothyroidism (P7) on replacement therapy, and autoimmune thyroiditis in euthyroidism not requiring therapy so far (P41). Adrenal insufficiency (all secondary to previous steroid therapy) was documented in 10 patients (23.8%).

None of the patients had diabetes mellitus. However, P17 experienced episodes of hyperglycemia. He underwent HSCT for XL-CGD with a complex pre-HSCT clinical history, including liver abscess, *Actinomyces* lung infection, and *M. chelonae* sepsis. To control the *M. chelonae* infection, he was initiated on IFN-γ therapy. During this period, he developed severe pancreatitis, requiring supportive care and surgical drainage. In the early post-HSCT phase, he exhibited intermittent hyperglycemia, which initially resolved spontaneously.

Following transplantation, he developed immunocomplex glomerulonephritis diagnosis, for which he received steroid therapy. He experienced steroid-induced hyperglycemia with glycosuria, which resolved upon tapering the steroids.

Currently, at 18 years of age and off immunosuppressive therapy, his latest assessment revealed recurrent glycosuria and borderline elevated glycated hemoglobin A1c levels. Therefore, he is scheduled for further evaluation as part of his transition to adult diabetes care.

### Skin and dental outcomes

Posttransplant cutaneous complications were observed in eight patients (19%), including late-onset (>2 years) herpes zoster infection requiring antiviral therapy in three patients, *M. chelonae* skin infection in one patient, persistent post-chemotherapy pigmentary changes were noted in two patients, and one experienced alopecia with no other cGvHD symptoms. Recurrent Bowen’s disease, associated with voriconazole-induced photosensitivity, was documented at 21.8 mo after HSCT in P28 (previously reported) (20). At the time of submitting this report, P31 developed localized soft tissue spindle cell sarcoma at the neck at 8 years after HSCT with positron emission tomography-CT scan showing no evidence of metastasis. Extended workup identified a necrotizing spindle-cell neoplasm with weak smooth-muscle-actin staining, granular cytoplasmic β-catenin expression, negative broad lineage and Epstein–Barr virus/human herpesvirus 8 immunostaining, and a high Ki-67 proliferation index. RNA fusion testing was negative, and methylation profiling favored undifferentiated sarcoma with low confidence. Data overall were consistent with undifferentiated spindle-cell sarcoma. Notably, the patient had sustained 100% donor engraftment and adequate immune reconstitution at the time of cancer development. Molecular assessment of the sarcoma is awaited. Mass was surgically removed.

Dental abnormalities were identified in six patients (14.3%). These included chronic gingival inflammation (*n* = 3), structural tooth abnormalities (*n* = 1), dental overcrowding (*n* = 1), and malocclusion (*n* = 1).

### Visual and audiologic outcomes

Visual impairment or recurrent ophthalmological complications were documented in six patients (14.3%). Cataract formation was observed in two patients (P11 and P17), with one case also having preexisting congenital glaucoma. One patient developed visual field defects as a sequela of pretransplant central nervous system infection. Herpes zoster ophthalmitis or keratitis affected two patients, while another patient was diagnosed with a choroidal naevus.

Audiological assessment revealed hearing impairment in seven patients (16.6%), with only one case demonstrating preexisting conductive hearing loss prior to transplantation. At last follow-up, five patients (P17, P21, P27, P28, and P41) demonstrated persistent hearing deficits, including four cases of sensorineural hearing loss requiring hearing aid support.

### Neurological outcome

Neurological complications were documented in six patients (14.3%). These manifestations included upper limb hypermobility affecting handwriting ability (P1), posttransplant epilepsy secondary to pre- and posttransplant cerebral aspergillosis (P11), frontal headache, and cognitive dysfunction following ADEM, associated with white matter changes on CT (P15), tremors attributed to thyrotoxicosis (P29), and persistent sensory peripheral neuropathy affecting hands and feet that developed 7 years after the resolution of Miller Fisher syndrome (P30). Moreover, P2 had chronic herpetic neuralgic pain in the periorbital region following varicella zoster infection. At last assessment (8 years after HSCT), he remained on Gabapentin therapy. At last follow-up, four patients (P2, P11, P15, and P30) continued to demonstrate persistent neurological symptoms.

### Neurocognitive outcome

Neurocognitive assessment at last follow-up identified abnormalities in six patients (14.3%). The spectrum of diagnoses encompassed autism spectrum disorder (ASD; P4, P5, P36, and P40), attention deficit hyperactivity disorder (ADHD; P27, P40), and learning disabilities (P37). All patients had a diagnosis of ADHD or ASD ahead of HSCT, with P27 having an identified 8p23 deletion syndrome that is known to be linked to ADHD.

### Psychological health outcome

Posttransplant psychological assessment revealed ongoing concerns in 19 patients (45.2%). The psychological manifestations included anxiety disorders (*n* = 9), needle phobia (*n* = 3), panic attacks (*n* = 1), and post-traumatic stress disorder (*n* = 1). Patients also experienced significant challenges with education (*n* = 7), sleep disturbances (*n* = 2), and social interactions (*n* = 5), including instances of bullying. One patient developed intermittent urinary incontinence. Among the nine patients who developed anxiety disorders, two cases (P40 and P41) were specifically associated with second therapeutic interventions, with these patients demonstrating increased anxiety specifically related to the additional procedures (P40 had episodes of short-term amnesia and visual hallucination together with anxiety). Nine patients (9/19, 47.3%) had a combination of psychological disturbances.

Within our cohort of patients, we did not observe an association between pre-HSCT autistic spectrum disorder, conditioning type (busulfan versus treosulfan), aGvHD (yes versus no), cGvHD (yes versus no), autoimmunity after HSCT (yes versus no), and any psychological outcome (details of the analysis are included in the supplementary document, Table S6).

## Discussion

We describe the long-term outcomes of 42 children with CGD who underwent HSCT between 1994 and 2020 and survived beyond 2 years after transplantation, with a median follow-up period of 8 years. Our cohort demonstrates excellent 10-year OS and EFS rates of 95.2% and 81%, respectively. Notably, survival was significantly higher in fully matched recipients and in the absence of cGvHD. Moreover, 10-year EFS was significantly higher in patients with full or mixed myeloid chimerism at 1-year after transplantation. These results align with previous multicenter studies evaluating HSCT outcomes in both pediatric and adult CGD cohorts (15, 17).

In our studied cohort, similar to Marsh et al. (21), we observed evidence for disease resolution in the majority of patients following HSCT, including resolution of inflammatory manifestations, particularly CGD-colitis. Nevertheless, a drop of myeloid chimerism can occur beyond 2 years after transplant being associated with disease recurrence, as seen in P10, or de novo CGD-related complications, as in P21 and P25, that stabilized after a second intervention. Therefore, long-term monitoring of myeloid chimerism is essential to be able to promptly diagnose and treat CGD-related manifestations.

Despite HSCT being a curative option, offering excellent long-term survival rates, we report a broad spectrum of late complications years after a successful HSCT that can significantly impact quality of life, emphasizing the need for lifelong surveillance.

Autoimmune complications emerged as a significant long-term complication, affecting 33.3% of patients. The clinical spectrum of autoimmune manifestations was broad, with more than half of these cases being either late onset (>2 years after HSCT) or suffering from recurrent/relapsing autoimmunity, and three patients developing >1 form of autoimmune disorder after HSCT. The most frequent manifestations were cytopenia, autoimmune thyroid disease, and autoimmune neuropathy. Our incidence was notably higher than the 3–10% reported in general primary immunodeficiency transplant populations by Holbro et al., Lum et al., and Sherer et al. (22, 23, 24), though lower than the 50% incidence observed in Yanir et al.’s CGD cohort of 24 patients, where alemtuzumab was administered at higher doses and closer to the procedure (25).

Lymphodepleting conditioning regimens together with unrelated grafts and the development of GvHD were found to be factors frequently present in patients that developed AIHA and autoimmune thyroid disease after HSCT (26, 27, 28).

In our cohort, similar to Yanir et al., we found no significant association between aGvHD, cGvHD, conditioning regimens, use of serotherapy, or mixed/low myeloid chimerism and the incidence of autoimmunity. However, the small sample size limits definitive conclusions. Similarly, no significant association was observed between delayed T cell recovery and the occurrence of autoimmunity (Table S5). Consistent with our findings, mixed myeloid or T cell chimerism, per se, does not imply a higher risk for secondary autoimmunity (24). The mechanisms underlying posttransplant autoimmunity in CGD remain incompletely understood. CGD-related chronic inflammation may disturb thymic regeneration and central tolerance after HSCT, reducing recent thymic emigrant output and skewing selection. The majority of our studied cohort had at least one, if not more than one, comorbidity, and 45% had colitis/fistulizing disease. Moreover, we noted that three patients had developed autoimmunity after a second intervention. Exposure to a second conditioned HSCT or DLI might have compromised thymic function leading to an impaired selection of autoreactive thymocytes and increased susceptibility to autoimmunity. Another possible explanation for autoimmunity might be attributed to the delayed T cell recovery leading to escape of autoreactive B cells, which would otherwise be prevented through central and peripheral tolerance. Moreover, Drozdov et al. reported that older age at transplantation, greater intensity of conditioning, and occurrence of grade 2–4 aGvHD were strongly associated with slower thymic-derived immune reconstitution (29). Chronic inflammation pre-HSCT or post-HSCT as explained might have contributed to the delayed immune recovery and increased rates of autoimmunity.

We have clearly observed delayed T cell recovery among our cohort of CGD patients with median time to reach CD4^+^ ≥300 and CD3^+^ ≥1,000 cells/μl being 10.3 and 14.4 mo among CGD in comparison to a cohort of 114 severe combined immunodeficiency who received the transplant at the same time interval in our center and had significantly earlier T cell recovery; CD4^+^ ≥300 and CD3^+^ ≥1,000 cells/μl being 3.9 and 7.2 mo, respectively, with lower rates of autoimmunity of 20% (unpublished data). Moreover, carriers of XL CGD, regardless of NADPH oxidase activity, have increased propensity for autoimmunity, including non-hematological autoimmunity (30); thus, a potential intrinsic defect might contribute to the higher rates of autoimmunity among CGD. This could be explained by increased activity of residual recipient antigen-presenting cells due to chronic infection that triggers autoimmunity (25).

Given these uncertainties, long-term follow-up with comprehensive hematological and clinical monitoring remains essential, maintaining a high index of suspicion for emerging autoimmune complications.

Endocrine dysfunction emerged as a prevalent long-term complication in our cohort, with significant implications for patient quality of life. We observed a high prevalence of gonadal dysfunction, which raises important concerns about future fertility. Short stature and adrenal insufficiency were frequently documented, requiring careful long-term management.

Metabolic disturbances were notably common, with 36% of patients classified as overweight or obese. While this partly reflects trends in the general pediatric population reported by NHS England, the implications for post-HSCT patients are potentially more severe due to their unique risk profile. In fact, these patients face additional risk factors for metabolic syndrome, including the effects of previous steroid therapy, reduced physical activity during treatment, and possible altered glucose metabolism after conditioning. The combination of these factors with obesity significantly increases the risk of long-term cardiovascular complications and insulin resistance, emphasizing the critical importance of early intervention through targeted lifestyle modification strategies (31). The high prevalence of endocrine complications in our cohort likely reflects the iatrogenic effects of conditioning regimens, particularly on the hypothalamic-pituitary axis and gonadal function. This risk is not unique to HSCT. Current CGD gene therapy protocols still rely on busulfan-based myeloablation and may therefore impact endocrine glands as well. Any reduction in toxicity will depend on future gene editing approaches with less toxic, targeted conditioning (32, 33, 34). However, until gene therapy becomes widely available, careful endocrine monitoring and proactive management pre-HSCT through testicular and ovarian cryopreservation and after HSCT through regular assessment and early intervention for cases with delayed puberty or gonadal dysfunction remain essential components of long-term post-HSCT care, with particular attention to growth, puberty, and fertility.

Bone health emerged as a particular concern, with a notably high prevalence of skeletal complications. While osteopenia was observed in 11.9% of patients, the significant finding was the high rate of non-osteopenic bone conditions (14.3%), including osteochondromas. This prevalence is particularly noteworthy when compared to our previous findings in the broader IEI post-HSCT population (35), where CGD patients were initially overlooked. The current analysis reveals that CGD patients may be at higher risk for non-osteopenic bone complications than initially recognized. These conditions often necessitate surgical intervention and can significantly impact growth and mobility, underlining the importance of regular skeletal monitoring in the post-HSCT period.

Psychological and neurocognitive dysfunction were reported in 59.5% of patients, including anxiety-related disorders and/or learning difficulties. Rare and chronic illnesses such as CGD have been shown to negatively impact mental health and quality of life in the long term (36, 37, 38, 39). Cole et al. demonstrated that CGD patients who underwent HSCT showed improved quality of life compared to those managed conservatively (38). However, the burden of the transplant procedure itself may exacerbate these long-term psychological sequelae, particularly when performed during developmental and educational years and in older children with a greater memory of the procedure (40). Currently, psychological support is mainly available during the pre-, peri-, and early posttransplant periods. Prospectively, we are working to implement standardized patient-reported outcomes at prespecified time points to systematize psychosocial assessment (40).

Our study has several important limitations. The exclusion of patients who died within the first 2 years after transplant introduces potential survival bias, particularly regarding the impact of donor source and pre-HSCT complications on outcomes. Due to the retrospective nature of the study, Karnofsky score was not systematically recorded. Additionally, our analysis lacks systematic quality of life assessments performed at regular intervals, which limits our ability to comprehensively evaluate the psychosocial impact of HSCT, particularly in relation to transplant age and subsequent complications. While our psychological outcome measures provide valuable insights into patient well-being, standardized quality of life assessments should be standard of care in long-term follow-up after HSCT for all IEIs. Furthermore, as a single-center study, our findings may not fully represent the broader global CGD population. Healthcare resource availability varies significantly between low-, middle-, and high-income settings, affecting access to donor options, conditioning regimens, timing of transplant, and comprehensive posttransplant care. Separately, geographical variations in pathogen epidemiology—such as higher prevalence of specific fungal species in certain regions or mycobacterial strains in various geographical areas—may result in distinct pre-HSCT morbidity patterns. These differences in healthcare infrastructure and region-specific disease manifestations likely influence both survival outcomes and the spectrum of post-HSCT complications across diverse global populations with CGD.

HSCT remains a curative therapeutic option for pediatric CGD patients, demonstrating excellent OS and EFS rates. However, the prevalence of long-term complications across multiple organ systems significantly impacts quality of life and necessitates ongoing vigilance. Our findings emphasize the critical importance of comprehensive, lifelong surveillance through structured multidisciplinary posttransplant programs. Future research should focus on understanding the interplay between long-term complications and psychological well-being, while exploring therapeutic strategies that might minimize long-term sequelae. Continued long-term follow-up studies will be crucial in optimizing patient outcomes and guiding therapeutic decisions for future generations of CGD patients.

## Materials and methods

### Study design

We retrospectively analyzed medical records of pediatric CGD patients who underwent allogeneic HSCT at GOSH NHS Foundation Trust between January 1, 1994, and December 31, 2020. Eligibility criteria included (i) confirmed diagnosis of CGD, (ii) survival for at least 2 years following the first HSCT, and (iii) no prior gene therapy. Diagnosis was established based on clinical presentation or family history consistent with CGD alongside abnormal neutrophil function tests assessed by dihydrorhodamine assay and/or NBT. Genetic testing and protein analysis were performed—in most cases—to confirm the diagnosis.

A total of 55 patients were identified, of whom 13 were excluded (Table S1): four had HSCT at University College London as adolescents (median age 16.4 years, range = 14.3–19.1 years), four had died within the first 2 years after HSCT, three were lost to follow-up before reaching the 2-year survival threshold, two had gene therapy as a first intervention. The final cohort consisted of 42 patients included in the analyses. Data were collected from electronic hospital records including clinic letters, laboratory results and imaging reports. Growth parameters were monitored and documented using pediatric centile charts.

Data collected included patients demographics (patient age, gender, CGD diagnosis, and age at HSCT), pretransplant comorbidities specifically focusing on organ involvement (pulmonary disease, CGD-associated colitis, perianal disease, hepatic complications, and central nervous system manifestations), transplant data (donor characteristics, stem cell source, and conditioning regimen specifications), in addition to GvHD prophylaxis, and rates of aGvHD and cGvHD (41, 42). Patients received either RIC or MAC protocols. RIC included treosulfan/fludarabine or fludarabine/melphalan or RIC busulfan (targeted area under the curve between 45 and 65 mg*h/L)/fludarabine. Myeloablative conditioning included myeloablative busulfan (targeted busulfan area under the curve >70 mg*h/L) in conjunction with either cyclophosphamide or fludarabine ± melphalan or combined treosulfan/fludarabine/thiotepa conditioning.

Late effects post-HSCT surveillance included 10-year OS, 10-year EFS, factors influencing 10-year OS and EFS, autoimmunity, kinetics of immune recovery, donor engraftment, and a comprehensive assessment of organ-specific late effects.

The study was conducted in accordance with Good Clinical Practice guidelines and the Declaration of Helsinki principles. It was registered as an audit activity at GOSH (registration number 4074). Written informed consent was obtained from all patients’ parents or legal guardians prior to study inclusion.

### Primary outcome measures

#### OS and EFS

OS and EFS were designated as the primary endpoints for this study. The date of last follow-up was determined as the final clinical review at GOSH prior to transition to adult services.

OS was defined as the time interval from initial HSCT to death from any cause. In the survival analysis, patients who were still alive at their last follow-up were censored at that time point, meaning their survival time was recorded as at least until that date, though their outcome remains unknown. EFS was defined as the time interval from initial HSCT to the first occurrence of any of the following events: death from any cause or requirement for secondary therapeutic intervention, including DLI, CD34^+^ stem cell top-up, gene therapy, or second conditioned transplant. Similarly, patients who had not experienced any events at their last follow-up were withdrawn from analysis at that time point.

### Secondary outcome measures

#### Chimerism analysis and immune recovery

Chimerism status was assessed using validated molecular techniques: fluorescence in situ hybridization or polymerase chain reaction analysis of short tandem repeats. Lineage-specific chimerism was evaluated in peripheral blood mononuclear cells and in specific cellular fractions: T lymphocytes (CD3^+^), B lymphocytes (CD19^+^), and granulocytes (CD15^+^). Donor chimerism was categorized as “full” (≥95%), “mixed” (25–94%), or “low” (<25%). Chimerism evaluations were recorded at time points 1, 3, 5, and 10 years after HSCT.

Immune reconstitution was monitored through flow cytometric analysis of lymphocyte subsets conducted at 6 mo, 1, 3, 5, and 10 year after HSCT, with additional evaluations performed as clinically indicated in cases of complications or sustained immunosuppression. T cell recovery kinetics were assessed—from the time of first transplant—by monitoring time to achieve specific cellular thresholds: CD3^+^ ≥1,000 cells/μl, CD4^+^ ≥300 cells/μl, and CD4^+^ ≥500 cells/μl.

Humoral immunity was evaluated through serial measurements of immunoglobulin levels and assessment of vaccine responses after HSCT. Specific antibody responses to tetanus and pneumococcal vaccines were measured and classified as normal, impaired, or absent based on reference ranges from healthy controls.

#### Autoimmune disease and organ-specific late effects

A structured approach to organ-specific surveillance was implemented across multiple systems. Pulmonary function was evaluated through standardized lung function testing, spirometry, DLCO, and chest CT imaging when clinically indicated. Gastrointestinal monitoring focused on both CGD colitis recurrence and new-onset gastrointestinal manifestations, while cardiac assessment was conducted through echocardiography. Liver health was monitored through routine enzyme profiling alanine transaminase, AST, and GGT) and ultrasound evaluation when abnormal.

Kidney function assessment included serum creatinine and albumin measurements, complemented by urinary albumin/creatinine and protein/creatinine ratios were abnormal.

Comprehensive endocrine surveillance included growth and metabolic monitoring, involving tracking of weight and height trajectories (31), lipid profiles, and glycemic indices. Bone health evaluation was undertaken through gait assessment, DEXA scanning for bone density, and additional radiological investigations as required. Gonadal function was evaluated through measurement of luteinizing hormone, follicle-stimulating hormone, estradiol, and testosterone levels, thyroid function through TSH and free T4 levels, and adrenal status through cortisol and adrenocorticotropic hormone.

Additionally, neurological evaluation, hearing, and vision screening were performed, along with monitoring of dermatological and dental health over time.

### Statistical analysis

Statistical analysis was performed using IBM SPSS Statistics software (version 28.0.0.0 [190], IBM Corporation). Continuous variables were presented as median values with ranges, while categorical variables were expressed as frequencies and percentages.

Survival analyses, including OS and EFS, were estimated using the Kaplan–Meier method. Comparisons between survival curves were performed using the log-rank test (Mantel–Cox). Given the limited size of the study cohort, descriptive statistics were predominantly employed to characterize the study population and outcomes. Statistical significance was defined as a two-sided P value <0.05.

All statistical figures were generated using IBM SPSS Statistics software, and additional scientific illustrations were created using BioRender (BioRender.com).

### Online supplemental material

Table S1 summarizes the characteristics of excluded CGD cases. Tables S2, S3, S4, S5, and S6 report exploratory analyses of factors not significantly associated with 10-year OS and EFS, non-osteopenic bone lesions, gonadal abnormalities, delayed T cell recovery and subsequent autoimmunity, and posttransplant psychological complications, respectively (all presented as numbers/percentages with corresponding P values).

## Supplementary Material

Table S1 shows the detailed characteristics of excluded CGD cases.

Table S2 shows the factors not significantly associated with 10-year OS and 10-year EFS (percentages and P values) calculated using the Kaplan–Meier method.

Table S3 shows the non-osteopenic bone lesions posttransplant and association with conditioning regimens, aGvHD, autoimmunity posttransplant, and use of systemic steroids (percentages and P values).

Table S4 shows the gonadal abnormalities posttransplant and association with conditioning regimens and gender (percentages and P values).

Table S5 shows the analysis of delayed T cell recovery at 6 mo after HSCT and its association with the development of autoimmunity (number and P values).

Table S6 shows the posttransplant psychological complications after HSCT and association with pre-HSCT ASD, conditioning regimens, aGvHD, cGvHD, and posttransplant autoimmunity (percentages and P values).

## Data Availability

The data are available from the corresponding author upon reasonable request.
