# Supplementary material for: Beyond survival: Multisystem long-term outcomes following HSCT in chronic granulomatous disease
Source: J Hum Immun. 2026 Feb 6;2(2):e20250076. doi: 10.70962/jhi.20250076 (PMC13177677; doi:10.70962/jhi.20250076)
Supplement: Table S1 — shows the detailed characteristics of excluded CGD cases. [file jhi_20250076_tables1.docx]

**Table S1.** Detailed characteristics of excluded CGD cases.

|  | **PATIENT CODE** | | | | | | | | | | | | |
| --- | --- | --- | --- | --- | --- | --- | --- | --- | --- | --- | --- | --- | --- |
|  | **1** | **2** | **3** | **4** | **5** | **6** | **7** | **8** | **9** | **10** | **11** | **12** | **13** |
| **Diagnosis** | AR (p47) | AR (p47) | XL | XL | XL | AR | XL | AR (p67) | XL | AR | XL | XL | XL |
| **Comorbidities** | Skin folliculitis MRSA, family history | Fungal lung infection, skin abscess | Colitis/  FTT | Colitis | Chest infection, granulomata in lungs | Pancolitis with perianal abscess | Perianal abscess, lymphadenopathies with granuloma, liver lesion suggestive of infections | Cerebral abscesses craniotomies X3 (Aspergillus), recurrent chest | Chest infections, skin granuloma, asymptomatic meningitis | Cervical abscesses | Chest infections, mouth ulcers, diarrhea | Aspergillo pneumonia | Liver and spleen abscess, poor weight gain, otitis, lymphadenitis |
| **Age at procedure** | 4y 3m | 4y 4m | 10y 8m | 15y 4m | 4y 7m | 17y 5m | 3y 3m | 10y 4m | 14y 3m | 14y 4m | 19y 1m | 9y 2m | 2y 6m |
| **HSCT or Gene therapy** | HSCT | HSCT | HSCT | HSCT | HSCT | HSCT | HSCT | HSCT | HSCT | HSCT | HSCT | Gene Therapy | Gene Therapy |
| **Donor type** | MUD | MUD | MUD | MSD | MUD | MUD | MUD | MMUD | MSD | MSD | MMUD | MMUD | / |
| **Transplant cells** | BM | BM | PBSC | BM | BM | / | BM | BM | BM | BM | BM | BM | / |
| **HLA** | 10/10 | 10/10 | 10/10 | 10/10 | 10/10 | 10/10 | 10/10 | 9/10 | / | 10/10 | 09/10 | 09/10 | / |
| **Conditioning Regimen** | Camp/  Flu/Bu | Camp/  Flu/Bu | Camp/  Flu/  Treo | ATG/  Flu/Bu | Camp/  Flu/Bu | Camp/  Flu/Bu | Camp/  Flu/Bu | Camp/  Flu/Bu | Bu/Flu | ATG/  Bu/Flu | Camp/  Flu/Bu | Bu/Flu/  Camp | / |
| **RIC/MAC** | RIC | RIC | RIC | RIC | RIC | / | RIC | RIC | / | / | RIC | RIC | / |
| **GVHD prophylaxis** | CSA/  MMF | CSA/  MMF | CSA/  MMF | CSA/  MMF | CSA/  MMF | CSA/  MMF | CSA/  MMF | / | / | / | CSA/  MMF | / | / |
| **Reason to be excluded** | Follow Up available <2y | Follow Up available <2y | Deceased before 2y post HSCT | HSCT in other centre | Deceased before 2y post HSCT | HSCT in other centre | Deceased before 2y post HSCT | Deceased before 2y post HSCT | Follow Up available <2y | HSCT in other centre | HSCT in other centre | Gene therapy as first procedure | Gene therapy as first procedure |

AR=autosomal recessive, ATG= Anti-thymocyte globulin, BM=bone marrow, Bu=busulfan, Camp=campath, CSA= cyclosporine, Flu=fludarabine, FTT=failure to thrive, HSCT= hematopoietic stem cell transplant, MAC=myeloablative conditioning, MMF= Mycophenolate mofetil, MMUD=mismatched unrelated donor, MSD=matched sibling donor, MUD=matched unrelated donor, PBSC=peripheral blood cells, RIC=reduced intensity conditioning, Treo= treosulfan, XL=X-linked.
