# Supplementary material for: Beyond survival: Multisystem long-term outcomes following HSCT in chronic granulomatous disease
Source: J Hum Immun. 2026 Feb 6;2(2):e20250076. doi: 10.70962/jhi.20250076 (PMC13177677; doi:10.70962/jhi.20250076)
Supplement: Table S2 — shows the factors not significantly associated with 10-year OS and 10-year EFS (percentages and P values) calculated using the Kaplan–Meier method. [file jhi_20250076_tables2.docx]

**Table S2.** Factors not significantly associated with 10-year OS and 10-year EFS (percentages and p-values) calculated using the Kaplan Meier method.

| **List of factors** | 10-year EFS | | 10-year OS | |
| --- | --- | --- | --- | --- |
|  | % | p-value | % | p-value |
| Age >5y vs <5y | 84.4 vs 70.0 | 0.194 | 96.9 vs 90.0 | 0.390 |
| CGD diagnosis XL vs AR | 79.3 vs 84.6 | 0.864 | 93.1 vs 100.0 | 0.362 |
| Colitis - Yes vs No | 76.5 vs 84.0 | 0.422 | 94.1 vs 96.0 | 0.707 |
| Lung disease - Yes vs No | 80.0 vs 81.8 | 0.923 | 100.0 vs 90.9 | 0.173 |
| Liver abscesses - Yes vs No | 75.0 vs 82.4 | 0.652 | 100.0 vs 94.1 | 0.518 |
| Perianal disease - Yes vs No | 75.0 vs 83.3 | 0.615 | 100.0 **vs** 93.3 | 0.345 |
| Stem cell source – BM vs PBSC vs Cord | 88.8 vs 66.7 vs 100.0 | 0.149 | 96.0 vs 93.3 vs 100.0 | 0.911 |
| Conditioning regimen – MAC vs RIC | 90.0 vs 78.1 | 0.339 | 100.0 vs 93.8 | 0.399 |
| Serotherapy - Yes vs No | 78.4 vs 100.0 | 0.188 | 94.6 vs 100.0 | 0.505 |
| aGvHD - Yes vs No | 87.0 vs 73.7 | 0.478 | 95.7 vs 94.7 | 0.852 |
| Autoimmunity in the first 2y post HSCT - Yes vs No | 100.0 vs 75.8 | 0.099 | 100.0 vs 93.9 | 0.442 |
| CD4 ≥300 vs. <300 at 6 months | 80.0 vs 83.3 | 0.915 | 100.0 vs 96.7 | 0.564 |
| CD4 ≥500 vs. <500 at 6 months | 66.7 vs 82.4 | 0.650 | 100.0 vs 97.1 | 0.752 |
| CD3 ≥1000 vs. <1000 at 6 months | 75.0 vs 82.1 | 0.858 | 100.0 vs 96.4 | 0.540 |

aGvHD=Acute Graft-versus-Host Disease, AR=autosomal recessive, BM=Bone Marrow, CGD=Chronic Granulomatous Disease, EFS=Event-Free Survival, HSCT=Hematopoietic Stem Cell Transplantation, MAC=Myeloablative Conditioning, OS=Overall Survival, PBSC=Peripheral Blood Stem Cells, RIC=Reduced-Intensity Conditioning, XL=X-linked.
