# Supplementary material for: Beyond survival: Multisystem long-term outcomes following HSCT in chronic granulomatous disease
Source: J Hum Immun. 2026 Feb 6;2(2):e20250076. doi: 10.70962/jhi.20250076 (PMC13177677; doi:10.70962/jhi.20250076)
Supplement: Table S3 — shows the non-osteopenic bone lesions posttransplant and association with conditioning regimens, aGvHD, autoimmunity posttransplant, and use of systemic steroids (percentages and P values). [file jhi_20250076_tables3.docx]

**Table S3.** Non-osteopenic bone lesions post-transplant and association with conditioning regimens, aGvHD, autoimmunity post-transplant and use of systemic steroids (percentages and p-values).

| **Non-osteopenic bone changes N=6/42** | **Conditioning Type (Yes=busulfan; No=treosulfan)** | **aGvHD** | **Post-HSCT autoimmunity** | **Systemic steroids** |
| --- | --- | --- | --- | --- |
| Yes | 5/28 (17.9%) | 2/25 (8.0%) | 1/14 (7.1%) | 1/11 (9.1%) |
| No | 1/13 (7.7%) | 4/17 (23.5%) | 5/28 (17.9%) | 5/31 (16.1%**)** |
| RR (Yes vs No) | 2.32 | 0.52 | 0.46 | 0.60 |
| CI (95%) | 0.30-17.92 | 0.16-1.66 | 0.07-2.91 | 0.09-3.87 |
| Fisher Test p-value | 0.645 | 0.202 | 0.489 | 0.999 |

aGvHD=Acute Graft-versus-Host Disease, CI=confidence interval, HSCT=Hematopoietic Stem Cell Transplantation, RR=Relative Risk.
