# Supplementary material for: Beyond survival: Multisystem long-term outcomes following HSCT in chronic granulomatous disease
Source: J Hum Immun. 2026 Feb 6;2(2):e20250076. doi: 10.70962/jhi.20250076 (PMC13177677; doi:10.70962/jhi.20250076)
Supplement: Table S4 — shows the gonadal abnormalities posttransplant and association with conditioning regimens and gender (percentages and P values). [file jhi_20250076_tables4.docx]

**Table S4.** Gonadal abnormalities post-transplant and association with conditioning regimens and gender (percentages and p-values).

| **Gonadal abnormalities N=9/41** | **Conditioning Type (Yes=busulfan; No=treosulfan)** | **Conditioning intensity (Yes=MAC; No=RIC)** | **Gender (Yes=male; No=female)** |
| --- | --- | --- | --- |
| Yes | 7/28 (25.0%) | 4/10 (40.0%) | 6/35 (17.1%) |
| No | 2/13 (15.4%) | 5/32 (15.6%) | 3/7 (42.9%) |
| RR (Yes vs No) | 1.63 | 2.56 | 2.50 |
| CI (95%) | 0.39-6.77 | 0.85-7.74 | 0.81-7.69 |
| Fisher test p-value | 0.692 | 0.181 | 0.155 |

CI=confidence interval, MAC=Myeloablative Conditioning, RIC=Reduced-Intensity Conditioning, RR=Relative Risk.
