# Supplementary material for: Beyond survival: Multisystem long-term outcomes following HSCT in chronic granulomatous disease
Source: J Hum Immun. 2026 Feb 6;2(2):e20250076. doi: 10.70962/jhi.20250076 (PMC13177677; doi:10.70962/jhi.20250076)
Supplement: Table S5 — shows the analysis of delayed T cell recovery at 6 mo after HSCT and its association with the development of autoimmunity (number and P values). [file jhi_20250076_tables5.docx]

**Table S5.** Analysis of delayed T cell recovery at 6 months post-HSCT and its association with the development of autoimmunity (number and p-values).

| **Autoimmunity anytime** | **N** | **CD3<1000/mmc** | **CD4<500/mmc** | **CD4<300/mmc** |
| --- | --- | --- | --- | --- |
| Yes | 14 | 11/12 | 14/14 | 11/14 |
| No | 28 | 17/24 | 23/26 | 19/26 |
| Total | 42 | 36 | 40 | 40 |
| p-value |  | 0.162 | 0.263 | 0.508 |
