# Supplementary material for: Beyond survival: Multisystem long-term outcomes following HSCT in chronic granulomatous disease
Source: J Hum Immun. 2026 Feb 6;2(2):e20250076. doi: 10.70962/jhi.20250076 (PMC13177677; doi:10.70962/jhi.20250076)
Supplement: Table S6 — shows the posttransplant psychological complications after HSCT and association with pre-HSCT ASD, conditioning regimens, aGvHD, cGvHD, and posttransplant autoimmunity (percentages and P values). [file jhi_20250076_tables6.docx]

**Table S6.** Post-transplant psychological complications post-HSCT and association with pre-HSCT autism spectrum disorder, conditioning regimens, aGvHD, cGvHD, and post-transplant autoimmunity (percentages and p-values).

| **Psychological complications after HSCT**  **N=19/42** | **Pre-HSCT ASD** | **Conditioning Type (Yes=busulfan; No=treosulfan)** | **aGvHD** | **cGvHD** | **Post-HSCT autoimmunity** |
| --- | --- | --- | --- | --- | --- |
| Yes | 4/6 | 14/28 | 13/25 | 2/6 | 5/14 |
| No | 15/36 | 5/13 | 6/17 | 17/36 | 14/28 |
| RR (Yes vs No) | 1.60 | 1.30 | 1.47 | 0.71 | 0.71 |
| CI (95%) | 0.81-3.17 | 0.60-2.84 | 0.70-3.11 | 0.22-2.30 | 0.32-1.58 |
| Fisher Test p-value | 0.384 | 0.524 | 0.353 | 0.673 | 0.515 |

ASD=autism spectrum disorder, aGvHD=Acute Graft-versus-Host Disease, cGvHD=Chronic Graft-versus-Host Disease, CI=Confidence Interval, HSCT=Hematopoietic Stem Cell Transplantation, RR=Relative Risk.
